# Changes in Serum Levels of Bone Morphogenic Protein 4 and Inflammatory Cytokines after Bariatric Surgery in Severely Obese Korean Patients with Type 2 Diabetes

**DOI:** 10.1155/2013/681205

**Published:** 2013-09-19

**Authors:** Mee Kyoung Kim, Eun-Hee Jang, Oak-Kee Hong, Hyun-Ji Chun, Soon-Jib Yoo, Ki-Hyun Baek, Wook Kim, Eung Kook Kim, Ki-Ho Song, Hyuk-Sang Kwon

**Affiliations:** ^1^Department of Internal Medicine, Yeouido St. Mary's Hospital, The Catholic University of Korea, 62 Yeouido-dong Youngdeungpo-gu, Seoul 150-713, Republic of Korea; ^2^Department of Internal Medicine, Hando Hospital, Seonbu 1-dong, Danwon-gu, Ansan 425-140, Republic of Korea; ^3^Department of Surgery, The Catholic University of Korea, Seoul, Republic of Korea

## Abstract

Serum bone morphogenic protein- (BMP-) 4 levels are associated with human adiposity. The aim of this study was to investigate changes in serum levels of BMP-4 and inflammatory cytokines after Roux-en-Y gastric bypass (RYGB). Fifty-seven patients with type 2 diabetes underwent RYGB. Serum levels of BMP-4 and various inflammatory markers, including high-sensitivity C-reactive protein (hsCRP), free fatty acids (FFAs), and plasminogen activator inhibitor- (PAI-) 1, were measured before and 12 months after RYGB. Remission was defined as glycated hemoglobin <6.5% for at least 1 year in the absence of medications. Levels of PAI-1, hsCRP, and FFAs were significantly decreased at 1 year after RYGB. BMP-4 levels were also significantly lower at 1 year after RYGB than at baseline (*P* = 0.024). Of the 57 patients, 40 (70%) had diabetes remission at 1 year after surgery (remission group). Compared with patients in the nonremission group, patients in the remission group had lower PAI-1 levels and smaller visceral fat areas at baseline. There was a difference in the change in the BMP-4 level according to remission status. Our data demonstrate a significant beneficial effect of bariatric surgery on established cardiovascular risk factors and a reduction in chronic nonspecific inflammation after surgery.

## 1. Introduction

Bone morphogenic proteins (BMPs) are members of the transforming growth factor-*β* superfamily. Although they were originally identified as bone-inducing proteins, the activities of BMPs are not restricted to bone formation [1]. Recent data indicate that BMP-4 induces the differentiation of white fat, the predominant type of adipose tissue [2]. Exposing pluripotent 10T1/2 cells to exogenous BMP-4 induces adipocyte lineage commitment [3]. Adipocyte precursor cells (APCs) derived from subcutaneous fat differentiate well in the presence of classical induction cocktail, whereas those from visceral fat differentiate poorly but can be induced to differentiate by addition of BMP-4 [4]. Activation of BMP-4 signaling may be associated with increased adiposity in humans [3]. It is still unclear whether BMP-4 acts via an endocrine mechanism or an autocrine pathway and whether cellular expression of BMP-4 reflects circulating blood levels of BMP-4 [5]. Recently, we showed that serum BMP-4 levels are associated with human adiposity, insulin resistance, and metabolic syndrome in nondiabetic subjects [6]. However, no studies have investigated the changes in BMP-4 levels in severely obese patients with type 2 diabetes after bariatric surgery. 

Adipose tissue is suggested to represent the major source of high-sensitivity C-reactive protein (hsCRP) and plasminogen activator inhibitor- (PAI-) 1. CRP plays a coordinating role by increasing the activity of PAI-1 in endothelial cells, thereby promoting thrombus formation [7]. The increase in PAI-1 levels may also result partly from the release of free fatty acids (FFAs) from fat tissues in diabetic patients, as well as in young nondiabetic individuals [7]. Bariatric surgery, especially Roux-en-Y gastric bypass (RYGB), causes significant rapid weight loss, including loss of a substantial amount of fat [8]. Our previous study showed a greater reduction of visceral fat than subcutaneous fat at 1 year after RYGB [9]. Changes in inflammatory cytokine levels after bariatric surgery have not been well characterized.

The aim of this study was to investigate changes in the serum levels of BMP-4 and inflammatory cytokines after RYGB in a severely obese population.

## 2. Subjects and Procedures

### 2.1. Study Subjects

We performed a prospective cohort study of severely obese Korean patients with type 2 diabetes who underwent laparoscopic RYGB at Yeouido St. Mary's Hospital between July 2009 and May 2010. Inclusion criteria for patients in this study were age 19–64 years, BMI > 30 kg/m^2^ (the recommended cutoff for bariatric surgery in Asians), and presence of type 2 diabetes that was inadequately controlled by conventional treatments [10, 11]. Exclusion criteria included history of type 1 or secondary diabetes, antiglutamic acid decarboxylase (GAD) antibody positivity, absence of *β*-cell function (fasting C-peptide concentration <1 ng/mL or glucagon-stimulated C-peptide concentration <1.5 ng/mL), and presence of severe complications of diabetes (proliferative retinopathy, serum creatinine >2 mg/dL, vascular complications, or severe neuropathy). Bypass surgery was performed by only two surgeons using the same laparoscopic technique in all patients. The operative technique produced a 15 to 20 cm^3^ gastric pouch, a 30 to 50 cm biliopancreatic limb, and a 100 cm Roux limb. Patients were seen at the diabetes-obesity clinic before surgery and at 1, 3, 6, 9, and 12 months after surgery. Our institutional ethics committee approved the study, and all patients provided written informed consent.

### 2.2. Anthropometric Measurements

Body weight and height were measured with the subject barefoot and wearing light clothing. These measures were used to calculate BMI as weight in kilograms divided by height in meters squared. A computed tomography (CT) scan at the L4-5 level was performed to measure the cross-sectional areas of total abdominal fat, visceral abdominal fat, and subcutaneous abdominal fat, using previously described methods. First, the total area of abdominal adipose tissue was measured at −190 to −30 Hounsfield units. The visceral fat area (VFA) was distinguished from the subcutaneous fat area (SFA) by manually tracing the abdominal muscular wall separating the two adipose tissue compartments. VFA was measured, and SFA was calculated by subtracting VFA from the total abdominal fat area. The abdominal fat CT scan was evaluated before and 12 months after bariatric surgery.

### 2.3. Definition of Remission of Diabetes

Remission was defined as a glycated hemoglobin (A1C) level <6.5% and a fasting glucose concentration <126 mg/dL for 1 year or longer without active pharmacological therapy [12].

### 2.4. Laboratory Analyses

Fasting glucose (8 h minimum fast) and A1C levels were measured in all patients at 3, 6, and 12 months after surgery. Glucose concentration was measured using the glucose oxidase method. The A1C level was measured using an automated HPLC analyzer (HLC-723 G7; Tosoh Corp., Tokyo, Japan). The serum C-peptide concentration was measured using an immunoradiometric assay (Institute of Isotopes Co., Ltd, Budapest, Hungary) before and 6 min after intravenous injection of 1 mg of glucagon. The serum BMP-4 level was measured using a Quantikine human BMP-4 immunoassay kit (R&D Systems, Minneapolis, MN, USA) with intra- and interassay coefficients of variation of 5.3 and 5.8%, respectively. The serum FFA level was measured by an enzymatic method (Daiichi Pharmaceutical Co., Tokyo, Japan). The PAI-1 level was measured using an enzyme-linked immunoassay (Diagnostica Stago, Asnières, France). Homeostatic model assessment of *β*-cell function (HOMA-*β*) was calculated from fasting glucose and fasting C-peptide values using the HOMA2 calculator.

### 2.5. Statistical Analyses

All data were analyzed using the SPSS statistical package (SPSS, Inc., Chicago, IL, USA). Data are presented as means ± SD unless stated otherwise. Logarithmic transformation was performed as necessary to achieve a normal distribution. Pearson correlational analysis was used to examine the relationships between inflammatory markers and various clinical parameters. Comparisons between before and 12 months after RYGB surgery were made using a paired *t-*test. Student's *t*-test and the *χ* ^2^ test were used to compare values between the remission and nonremission groups. Values of *P* < 0.05 indicated significance.

## 3. Results

### 3.1. Clinical Characteristics of Study Subjects

Data from 57 patients were analyzed (Table 1). All patients were treated preoperatively with oral hypoglycemic agents or insulin. The mean age of the patients was 43.5 ± 10.1 years, and the mean BMI was 34.2 ± 4.1 kg/m^2^. The mean duration of diabetes was 5.3 ± 3.9 years, and the mean A1C level was 8.6 ± 1.6%.

### 3.2. Correlation between Inflammatory Markers and Clinical Parameters

The preoperative BMP-4 level was positively associated with HOMA-*β* (*γ* = 0.677, *P* = 0.006). The BMP-4 level was positively, but nonsignificantly, correlated with serum levels of PAI-1 (*γ* = 0.471, *P* = 0.098), hsCRP (*γ* = 0.427, *P* = 0.087), and stimulated C-peptide (*γ* = 0.366, *P* = 0.135). The BMP-4 level was not correlated with BMI (*γ* = −0.074, *P* = 0.803) or VFA (*γ* = 0.108, *P* = 0.713). The preoperative serum PAI-1 level was positively correlated with the preoperative HbA1C level (*γ* = 0.378, *P* = 0.018), VFA (*γ* = 0.518, *P* = 0.001), and visceral-to-subcutaneous fat ratio (*γ* = 0.307, *P* = 0.064). 

### 3.3. Changes in Inflammatory Marker Levels after RYGB

**Figure 1:**
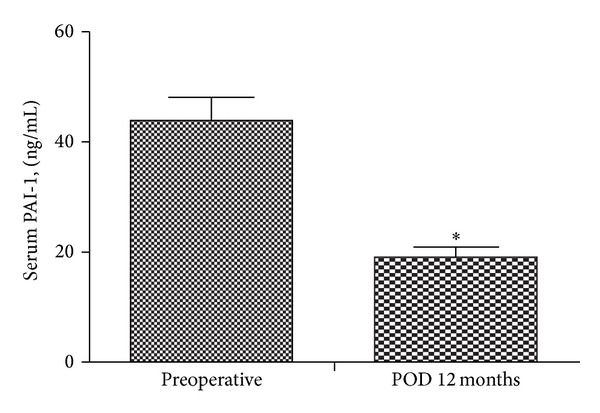
Changes in serum PAI-1 levels 12 months after bariatric surgery (*P < 0.05).

**Figure 2:**
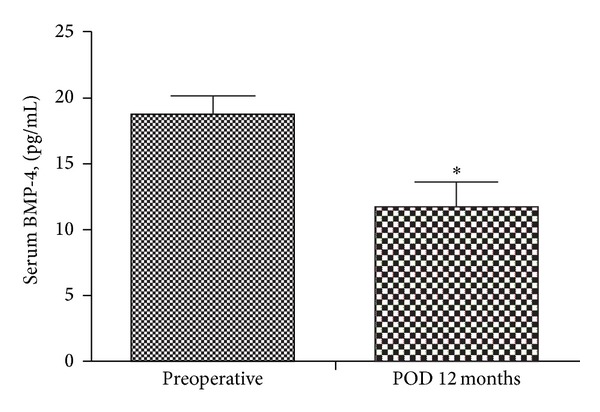
Changes in serum BMP-4 levels 12 months after bariatric surgery (*P < 0.05).

There were remarkable improvements in inflammatory marker levels, with significant decreases in the levels of PAI-1 (from 45.57 ± 25.0 to 21.37 ± 13.0 ng/mL, *P* < 0.001; Figure 1), hsCRP (from 14.52 ± 17.0 to 0.59 ± 0.6 mg/L, *P* < 0.001), and FFAs (from 850.0 ± 331.9 to 473.53 ± 188.7 *μ*Eq/L, *P* < 0.001) at 1 year after RYGB. BMP-4 levels were also significantly lower at 1 year after RYGB compared with baseline (18.3 ± 7.3 versus 12.7 ± 7.2 pg/mL, *P* = 0.024; Figure 2).

### 3.4. Comparison between Patients with Remission of Diabetes and Those with No Remission

Of the 57 patients, 40 (70%) had diabetes remission at 1 year after surgery. Compared with patients in the nonremission group, patients in the remission group had a shorter duration of diabetes (4.3 ± 3.6 versus 7.6 ± 3.9 years, *P* = 0.003), higher stimulated C-peptide level (10.2 ± 6.7 versus 7.0 ± 4.4 ng/mL, *P* = 0.041), and lower preoperative A1C level (8.34 ± 1.45 versus 9.22 ± 1.73%, *P* = 0.051; Table 2). At baseline, compared with patients in the nonremission group, patients in the remission group had a lower PAI-1 level (37.7 ± 20.7 versus 58.2 ± 27.2 ng/mL, *P* = 0.012), lower VFA (169.2 ± 51.6 versus 215.7 ± 53.3 cm^2^, *P* = 0.003), and lower visceral-to-subcutaneous fat ratio (0.53 ± 0.26 versus 0.79 ± 0.29, *P* = 0.002). 

There was a borderline significant difference in the change in the BMP-4 level according to remission status. In the remission group, BMP-4 levels tended to decrease at 12 months after surgery compared with baseline values (18.7 ± 5.1 versus 11.7 ± 7.3 pg/mL, *P* = 0.05; Table 2). In the nonremission group, the baseline and 12-month BMP-4 levels were not significantly different. The change in serum BMP-4 level differed between the remission and the nonremission groups (ΔBMP-4, −44.6 ± 13.8 versus 3.8 ± 24.8%, *P* = 0.086). 

## 4. Discussion

We found that the levels of the inflammatory markers PAI-1, hsCRP, and FFAs were decreased significantly 1 year after bariatric surgery in severely obese patients with type 2 diabetes mellitus. The serum BMP-4 level was decreased after RYGB, especially in the remission group and tended to be positively correlated with serum PAI-1 and hsCRP levels. Our data suggest that the serum levels of BMP-4 and other inflammatory markers such as PAI-1 and hsCRP decreased in response to weight loss after bariatric surgery.

BMP receptor 1A (BMPR1A) is one of the three specific BMP receptors that are essential for bone morphogenic protein signalling. In one study, visceral and subcutaneous BMPR1A mRNA expression levels were significantly higher in overweight and obese individuals compared with lean subjects, and BMPR1A mRNA expression correlated strongly with measures of obesity, including BMI, percentage body fat, and waist-to-hip ratio [13]. Because BMP-4 uses predominantly BMPR1A as its receptor, these results suggest that BMP-4/BMPR1A signaling may be involved in the pathogenesis of human obesity. In our previous study, the serum BMP-4 level was associated with obesity and the presence of metabolic syndrome [4]. In the present study, the serum BMP-4 level decreased after profound weight loss due to bariatric surgery.

As an inflammatory mediator in the vascular endothelium, BMP-4 is responsive to disturbed blood flow, increased oxidative stress, and inflammation [6]. Increased BMP activity enhances atherosclerosis and vascular calcification. High glucose levels strongly promote BMP activity in endothelial cells [14]. Hyperglycemia and diabetes activate vascular BMP activity, and this is instrumental in promoting vascular calcification, which may be limited by increasing BMP inhibition. Serum levels of BMP-4 increased progressively in rats transgenic for human islet amyloid polypeptide (HIP rats, a model of type 2 diabetes), resulting in a greater ability to induce BMP-dependent osteogenesis in calcifying vascular cells [14]. Persistently high levels of BMP-4 in vascular tissues or serum may be an early sign of diabetes [14]. In our study, serum BMP-4 levels decreased after RYGB, especially in the remission group, where the BMP-4 level was significantly decreased at 12 months after surgery compared with the baseline value. It is possible that the decrease in serum BMP-4 after bariatric surgery was mediated by amelioration of hyperglycemia in the remission group. Moreover, BMP-4 is a potential mediator of cardiovascular disease risk reduction after bariatric surgery.

PAI-1 is secreted mainly from visceral adipocytes and acts as an inhibitor of plasma fibrinolytic activity [15]. Impaired fibrinolysis can increase thrombosis and is therefore closely linked to the risk for cardiovascular disease. Reducing BMI in obese children has a favorable effect on the fibrinolytic system because of a decrease in PAI-1 levels [16]. In the present study, the preoperative serum PAI-1 level was closely related to VFA, and there was a significant decrease in PAI-1 at 12 months after surgery. The postoperative reduction in the PAI-1 level might have been caused by the reduction in body weight and visceral fat after surgery and may contribute to a lower cardiovascular mortality [15]. 

Compared with patients in the nonremission group, patients in the remission group had lower PAI-1 levels and VFA values at baseline. In our previous study, predominant visceral fat depots were a negative predictor of diabetes remission 1 year after bariatric surgery [9]. VFA remained higher in the nonremission group than in the remission group at 12 months after surgery. Serum PAI-1 levels at 12 months after surgery were also higher in the nonremission group than in the remission group. The change in the PAI-1 level after surgery was quite similar to the change in visceral fat depots. In the present study, patients with a shorter duration of diabetes, a higher C-peptide level, and a lower preoperative A1C level were more likely to achieve remission after RYGB. These results are consistent with a previous study showing that diabetes severity is an important predictor of remission [17]. According to a large Taiwanese study, diabetes remission, defined as a glycated hemoglobin level of ≤6%, was achieved in 70% of subjects at 12 months after bariatric surgery, and the glycemic response to gastric bypass was related to BMI (>35 kg/m^2^), duration of diabetes (<4 years), fasting C-peptide level (>2.9 ng/mL), and weight loss [17]. Failure of diabetes remission may result from a lack of patient compliance, inadequate weight loss, longstanding uncontrolled diabetes, or the disease in fact being type 1 diabetes (latent autoimmune diabetes in adults; LADA) [18].

Our study has some limitations. First, our study was limited by its relatively small sample size. Second, we did not perform oral glucose tolerance test to determine the remission of diabetes after surgery.

In conclusion, the serum levels of BMP-4 and inflammatory markers such as PAI-1 and hsCRP decreased significantly after RYGB in severely obese patients with type 2 diabetes mellitus. There were significant differences in the PAI-1 level and the change in the BMP-4 level according to remission status. Our data demonstrate a significant beneficial effect of bariatric surgery on established cardiovascular risk factors and a reduction in chronic nonspecific inflammation after surgery. 

## Figures and Tables

**Table 1:** Clinical characteristics of study subjects.

*N*	57
Age, years	43.5 ± 10.1
Sex, men/women	19/38
DM duration, years	5.3 ± 4.0
HbA1C, %	8.6 ± 1.6
BMI, kg/m^2^	34.2 ± 4.1
Body weight, kg	92.6 ± 16.4
Subcutaneous fat area, cm^2^	344.0 ± 119.2
Visceral fat area, cm^2^	182.7 ± 55.8
Fasting C-peptide, ng/mL	6.9 ± 4.4
Stimulated C-peptide, ng/mL	9.3 ± 6.3
Treatment, *n* (%)	
Oral hypoglycemic agents	35 (61%)
Insulin	22 (39%)

**Table 2:** Comparison of clinical characteristics of severely obese Korean subjects with type 2 diabetes between remission and nonremission groups.

	Remission (*n* = 40)			Nonremission (*n* = 17)		
	Preoperative	12 months	*P* value	Preoperative	12 months	*P* value
Fasting blood glucose, mg/dL	177.3 ± 40.7*	104.0 ± 12.9	<0.001	203.1 ± 66.1	149.8 ± 51.0	<0.001
HbA1c, %	8.3 ± 1.5*	5.7 ± 0.4	<0.001	9.2 ± 1.7	7.3 ± 0.9	<0.001
BMI, kg/m^2^	34.4 ± 4.5	26.1 ± 4.1	<0.001	33.2 ± 3.4	25.8 ± 3.1	<0.001
Visceral fat area, cm^2^	169.2 ± 51.6*	66.8 ± 35.5	<0.001	215.7 ± 53.3	101.3 ± 33.6	<0.001
Subcutaneous fat area, cm^2^	360.5 ± 122.4	212.8 ± 122.7	<0.001	303.8 ± 103.6	193.2 ± 74.3	<0.001
Visceral to subcutaneous fat area ratio	0.53 ± 0.26*	0.37 ± 0.22	<0.001	0.79 ± 0.29	0.57 ± 0.18	0.012
PAI-I, ng/mL	37.7 ± 20.7*	18.5 ± 8.2	<0.001	58.2 ± 27.2	23.6 ± 13.5	<0.001
FFA, *µ*Eq/L	844.1 ± 346.1	462.4 ± 164.0	<0.001	863.5 ± 306.5	494.4 ± 232.4	0.001
hsCRP, mg/L	15.3 ± 17.8	0.52 ± 0.43	<0.001	12.9 ± 15.7	0.74 ± 0.81	0.009
BMP4, pg/mL	18.7 ± 5.1	11.8 ± 7.3	0.054	17.8 ± 9.8	13.9 ± 7.4	0.564

*P < 0.05 when compared with those of preoperative in the nonremission group.
